# Case Report: Impact of dolphin kick implementation during backstroke finishes on swimming performance. From regional to olympic-level swimmers. A comparative case study

**DOI:** 10.3389/fspor.2025.1531427

**Published:** 2025-02-14

**Authors:** Konstantinos Papadimitriou, Stefanos Zafeiriadis, Nikos Papadimitriou, George Tsalis

**Affiliations:** ^1^ Faculty of Sport Sciences & Physical Education, Metropolitan College, University of East London, Thessaloniki, Greece; ^2^ Swimming Coach, Physical Education Teacher, Former Laboratory of Swimming Assistant Professor, Thessaloniki, Greece; ^3^ Department of Accounting and Finance, International Hellenic University, Sindos, Greece; ^4^ School of Physical Education and Sports Science, Aristotle University of Thessaloniki, Serres, Greece

**Keywords:** Classic, Dive–One hand, Dive–Streamline, regulations, regional, high-level

## Abstract

According to the World Aquatics (WA) an updated swimming regulation (SW) concerning the backstroke finish has been implemented, allowing, the swimmers to fully submerge their bodies at once when some part of their head pass the 5-meter mark immediately before touching the wall. Therefore, the present comparative case study aims to apply the new regulation on backstroke finish, examining the swimming efficiency and underwater kinematics of an elite Olympic-level swimmer, and comparing the data with the published ones from a previous study on regional-level swimmers. A 19-year-old male Olympic-level backstroke swimmer performed three all-out 20 m backstroke swims: (i) touching the wall, breaking the water's surface with one part of their body (Classic), (ii) touching the wall with one hand, submerging the body two strokes after passing the last 5 m (Dive–One Hand) and (iii) touching the wall on streamline position, submerging the body two strokes after passing the last 5 m (Dive–Streamline) Kinematic analysis of the intermediate 10 m and the last 5 m was conducted using a digital video camera. Descriptive statistics and the Crawford-Howell *t*-test were utilized for the comparisons between elite and regional-level swimmers. Based on the swimmer's analysis, intermediate 10 m were faster than that in regional level swimmers (Elite vs. Regional: 1.68 ± 0.1 vs. 1.29 ± 0.1 m·s^−1^, *p* = 0.00). Also, the elite-level swimmer increased his transition swimming speed (SS) (from 10 to the last 5 m) in all backstroke finishes (0.05 ± 0.03 vs. −0.07 ± 0.03 m·s^−1^). Also, in the last 5 m showed an extreme increase in velocity at the sink- to-finish speed (SFS), 2.16 and 2.28 m·s^−1^ for Dive–One hand, and Dive–Streamline, respectively. However, the tendency in velocity between backstroke finishes was similar between regional and elite-level swimmers. The elite swimmer can utilize the two variations of backstroke finishes more effectively compared to the regional-level swimmers. The swifter dolphin kick ability appears to play a significant role in achieving a successful backstroke finish, However, further investigations involving elite swimmers, differentiating the sinking approach and improving the speed before sinking (SBS) factor, could potentially provide more insights.

## 1 Introduction

Swimming performance is influenced by several physiological (1, 2), psychological (3, 4), coaching (5, 6), technique (7), strategy (8), and regulation factors (9). In 2023, World Aquatics announced an updated swimming regulation (SW) regarding the backstroke finish. According to this new rule (SW 6.3), swimmers can fully submerge their body once some part of their head passes the 5-m mark immediately before touching the wall to finish the race. Consequently, swimmers may potentially initiate dolphin kicks immediately after fully submerging their body. Therefore, swimmers can finish either by (i) placing one hand up (Dive–One hand) or (ii) assuming a streamlined position with both hands (Dive–Streamline), until they touch the wall on both occasions. This is in contrast to the previous, but still active, regulation in which some part of the swimmer had to break the water's surface during the touch on the wall (Classic) (9).

Dolphin kicks are the simultaneous utilization of vertically oriented motions of the feet (10–15) and their incorporation into competitive swimming presents a direct consequence of the proven reduction in wave drag through underwater swimming (15, 16), contributing to faster and more efficient movement (17). The utilization of dolphin kicks is present in the start and turn phases of a swimming event, and they have significant importance to its result, influencing swimmers' performance.

Suito et al. (18), Ikuta et al. (19), and Marinho et al. (20) stated that the stroke and turn technique, and start and turn phases, respectively, have greater influence than the finish on the race result. However, Marinho et al. (20) referred to the influence of the finish on performance in all swimming styles for 100 and 200 m events. They found that the start, specifically the underwater phase, and the turn, particularly the time slowing down between reaching the 45 m mark and touching the wall, as well as the slowdown between the wall touch and reaching the 15 m mark, had a greater influence on the performance of the events. The finish had a lesser influence on performance impairment and was predominantly negative in the 100 m breaststroke (in females), butterfly, and backstroke. In the 200 m, this influence was observed only in the butterfly.

Therefore, the examination of the backstroke finish, especially with the importance of dolphin kicks, has great interest. Papadimitriou and Papadimitriou (8) published a study examining and comparing these rule modifications in backstroke finishes in regional-level swimmers. According to the results, it was found that the Classic and Dive–One hand finish were faster than the Dive–Streamline finish in the last 5 m before the touch on the wall. On the other hand, only the Classic finish allowed the swimmers to maintain their swimming speed (SS) during the transition from the 10 m mark to the last 5 m. Another considerable point was the high correlation between the SS of dolphin kicks ability and the SS of both backstroke finishes (Dive–One hand and Dive–Streamline). According to this result, it was hypothesized that elite-level swimmers, who have faster dolphin kicks than national or regional-level swimmers (8), could potentially benefit from these types of backstroke finishes.

Therefore, the present study aims to examine the new regulation for backstroke finish by recording the swimming performance, efficiency, and underwater motion of an elite Olympic-level swimmer. Additionally, we aim to compare the data and their tendency with the already published data from the regional-level swimmers that we studied in a previous study. The research hypothesis was that the elite swimmer would have faster dolphin kick ability and SS during the underwater phase of the finishes compared to the regional-level swimmers. However, the intermediate SS (transition from 10 to the last 5 m before the finish) will be maintained only in the Classic backstroke finish.

## 2 Materials and methods

### 2.1 Participant

A 19-year-old male, Olympic-level, backstroke swimmer with a training experience of 10 years and 883 (64) WA points (9) was participated in the study. The swimmer was selected considering his participation in the Paris 2024 Olympic Games, his ability to swim the three backstroke distances (50–200 m), and the higher level than the regional swimmers who participated in the previous study.

According to the elite swimmer's anthropometrics and body composition measurements, a height of 1.73 m, weight of 67.1 kg, free fat mass (FFM) of 64.1 kg, fat mass (FM) of 3 kg, and body mass index (BMI) of 22.4 kg·m^2^ [mean ± standard deviation (SD) in parentheses] were recorded. The swimmer underwent training six days a week for approximately three and a half hours, comprising eleven sessions (nine focused on swimming and two on dryland training), covering a weekly distance of 46,550 (5,170) m. Throughout the measurement period, the swimmers engaged in a combination of high-volume and moderate-intensity water-based training, while the dryland training focused on plyometrics and elastic strength exercises.

Before the measurements, the swimmer and his coach were informed about the study's process and safety. Then, they both signed a written consent form to ensure the swimmer's participation. Also, the study was in accordance with the Declaration of Helsinki, and the design was approved by the Collaborative Research Ethics Committee (CREC) of the Metropolitan College (University of East London, approval number, 3451).

### 2.2 Procedure

The swimmer performed one 20 m dolphin kick and three all-out 20 m backstroke swims (one for each finishing type) with 10-min intervals between each attempt. The distance was split into three analysis points: the first 5 m was used for the swimmer's acceleration, the intermediate 10 m for the analysis of swimming efficiency (SI), and the last 5 m for the analysis of the backstroke finish (8).

**Figure 1:**
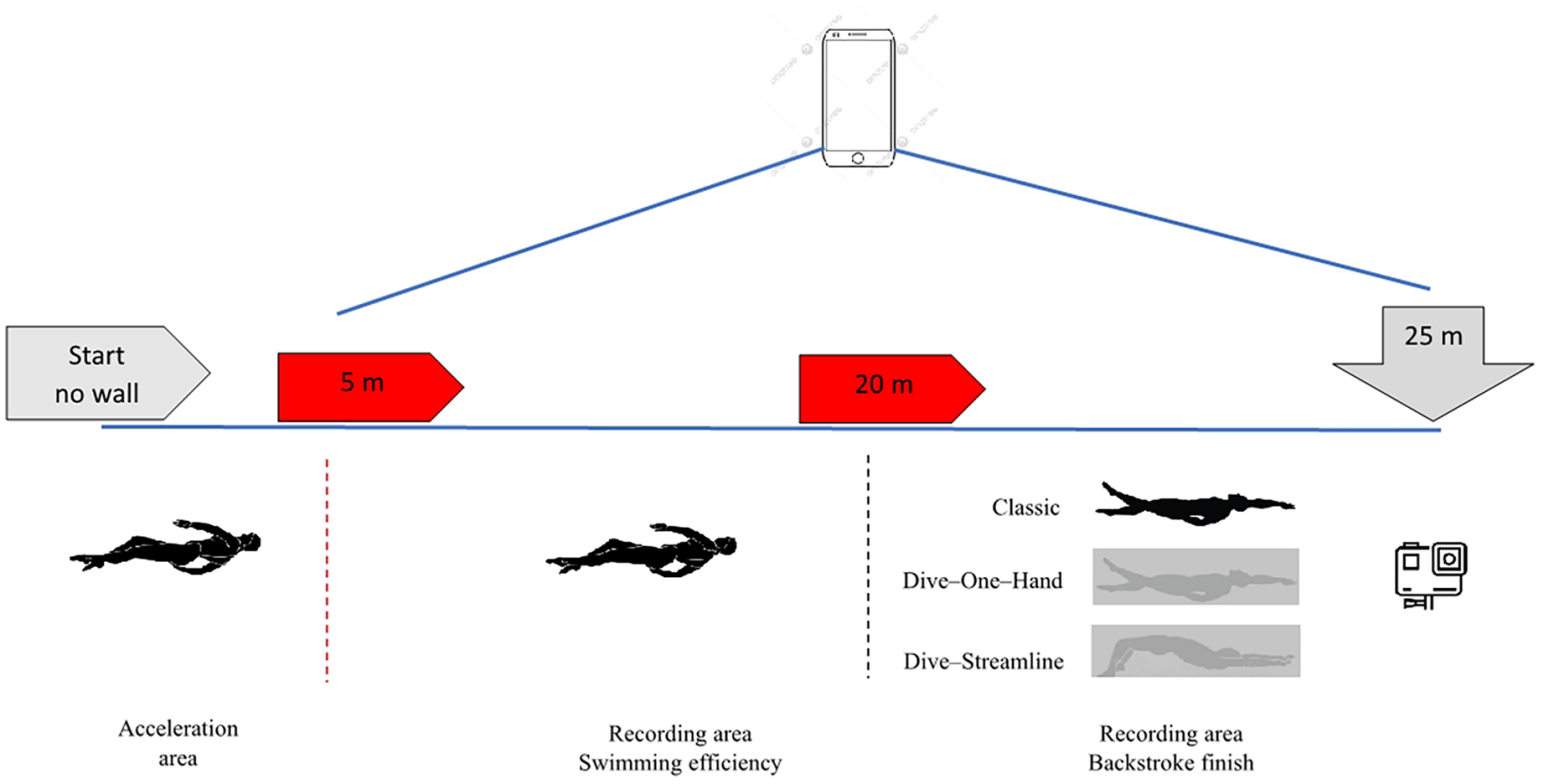
Depiction of measurements procedure.

Specifically, the kinematic assessment for backstroke began after the swimmer's head had passed the 5 m acceleration line finish and was completed when the swimmer's head had passed the finish line of intermediated 10 m. Also, the analysis of backstroke finish performance began when the swimmer's head had passed the finish line of the intermediated 10 m, continued for the last 5 m of the finish, and completed when the swimmer touched the wall with his hand (Figure 1).

### 2.3 Measurement tools

#### 2.3.1 Camera placement

An iPhone 15 Pro Max (Apple Inc. Foxconn, Cupertino, California) and GoPro Hero 9 (Woodman Labs, Inc., San Mateo, California) were used to film the swimmer's dolphin kicks and backstroke finishes. One camera was positioned outside and stably near the center of the 15 m (7.5 m) mark at a height of 3 m and with a 20 m distance between the camera and the swimmer, recording his performance in the sagittal plane (8, 21, 22), whereas the other camera was placed underwater (attached to the pool wall) to capture all attempts in the coronal plane (16). The measurement spots were calibrated with the Measure app of the iPhone 15 Pro Max (Apple Inc. Foxconn, Cupertino, California). Both cameras were synchronized during the measurements, with the moment when the swimmer's head had passed the 20 m mark as a starting point. The cameras captured footage at a rate of 60 frames per s (fps) (23, 24). During the swimmer's warm-up, two experts approved the cameras' function and sharpness before the measurements. The measurements were conducted in an indoor 50 m pool with water and air temperatures of 26 and 18 °C, respectively, and an average humidity of 60%.

#### 2.3.2 Kinematics and efficiency parameters

The kinematic analysis for the dolphin kicks swimming speed (SS) in the intermediate 10 m consisted of parameters such as swimming speed (SS) (distance/time) (m·s^−1^), stroke length (SL) (distance/strokes) (m/stroke), stroke rate (SR) [(time of 3 cycles/3) × 60] (strokes·min^−1^), and efficiency, calculating stroke index (SI) (speed × SL) (m^2^·s^−1^) (5, 8, 24). For the last 5 m, the following were computed: clear underwater distance (UD) (m), the SS in the last 5 m, and the velocity from the 5 m line until the moment before the sink [speed before sink (SBS)], calculating the velocity from the moment when the head passed the 5 m line until the moment before its sink, and the sink-to-finish velocity [Sink to Finish Speed (SFS)], which recorded the speed from the end of SBS until the touch of the wall with the hand. The depth (cm) of the head during the last stroke [Head depth (HD)] was also recorded, computing the displacement of the head from the highest to the lowest recorded spot during the sinking (before, during, and at the end of sinking). Additionally, the time (s) of this process was recorded [Time of head depth (THD)] (8, 16, 25). The analysis of the above-mentioned factors was conducted through the recorded videos by a coach with 12 years of experience, who assessed the swimmer's backstroke efforts by reviewing videos and recording their performance and efficiency indices (8) with the contribution of Kinovea, a video annotation tool designed for sport analysis, avoiding features with low accuracy (26).

#### 2.3.3 Observing the tendency between efforts in different level swimmers

Also, in the study, the descriptive results from 30 regional-level swimmers, WA points ≥300 (9 boys and 21 girls), aged 13.4 (1.0) years old, height 1.69 (0.05) m, weight 54.0 (4.9) kg, body mass index (BMI) 18.6 (0.8) kg·m^2^, and training experience of 7.7 (1.5) years, who participated in Papadimitriou and Papadimitriou's (2) study and have been already published, were examined, observing the kinematics tendency of the three backstroke finishes between the elite and the regional swimmers.

### 2.4 Statistical analysis

Descriptive statistics were presented as means and percentages (%), and the Crawford-Howell *t*-test (27) was used to compare a case with the mean of the previous study sample, with statistical significance set at *α* ≤ 0.05. The analysis was conducted with the software SPSS, Version 25.0 (Armonk, NY, USA: IBM Corp).

## 3 Results

### 3.1 Kinematic variables of elite swimmer

According to the studied indexes, the 15 m underwater dolphin kick velocity had a value of 1.83 m·s^−1^, while the SS, SL, SR, and SI were similar in the intermediated 10 m. However, the SS in the last 5 m was 1.7 and 5.1% faster in Classic compared to the Dive–One hand and Dive–Streamline finishes, respectively. Also, the transition SS (from 10 to the last 5 m) was increased more in the classic type by 5.6%, while in Dive–One hand and Dive–Streamline finishes, SS was increased by 2.9 and 0.6%, respectively (Table 1).

**Table 1:** Elite swimmer's intermediate (10 m) and finish (5 m) performance, efficiency, and kinematic variables between the three types of finish.

Type of finish	Intermediate 10 m					Last 5 m					
	SS (m·s^−1^)	SL (m)	SR (strokes·min^−1^)	SI (m^2^·s^−1^)	UD (m)	SS (m·s^−1^)	SN (n)	SBS (m·s^−1^)	SFS (m·s^−1^)	HD (cm)	THD (s)
Classic	1.67	0.90	68.60	3.03	—	1.77	4	—	—	—	—
Dive–One hand	1.69	0.90	69.40	3.08	4.10	1.74	1	0.97	2.16	24.19	0.27
Dive–Streamline	1.67	0.90	70.00	3.03	4.10	1.68	1	0.77	2.28	20.79	0.73
Df	−0.02	0.00	−0.80	−0.05	—	0.02	3	—	—	—	—
	0.00		−1.40	0.00	—	0.08	3	—	—	—	—
	0.02		−0.60	0.05	0.00	0.06	0	0.22	−0.12	3.40	0.46

SS, swimming speed; SL, stroke length; SR, stroke rate; SI, stroke index; UD, underwater distance; SBS, speed before sink; SFS, sink to finish speed; HD, head depth; THD, time of head depth; Df, difference between Classic and Dive–One hand, Classic & Dive–Streamline, and Dive–One hand & Dive–Streamline.

### 3.2 Kinematic variables of regional and elite level swimmers

The intermediate (10 m) indices of performance and efficiency parameters of the elite swimmer were faster or higher than the mean values of the regional-level swimmers (Table 2).

**Table 2:** Intermediate performance and efficiency variables between the regional and elite level swimmers in the three types of finish.

	Classic			*P*-value	Dive–One hand			*P*-value	Dive–Streamline			*P*-value
	Regional	Elite	Df		Regional	Elite	Df		Regional	Elite	Df	
SS (m·s^−1^)	1.30	1.67	−0.37	0.01*	1.30	1.69	−0.39	0.01*	1.27	1.67	−0.40	0.01*
SL (m)	0.82	0.90	−0.12	0.36	0.84	0.90	−0.06	0.43	0.82	0.90	−0.12	0.51
SR (strokes·min^−1^)	68.42	68.60	−0.18	0.00*	71.80	69.40	2.40	0.00	70.09	70.00	0.09	0.60
SI (m^2^·s^−1^)	2.14	3.03	- 0.89	0.08	2.15	3.08	−0.93	0.12	2.15	3.03	−0.88	0.14

SS, swimming speed; SL, stroke length; SR, stroke rate; SI, stroke index; Df, Difference between regional and elite level swimmers.

* Statistical significant difference.

**Figure 2:**
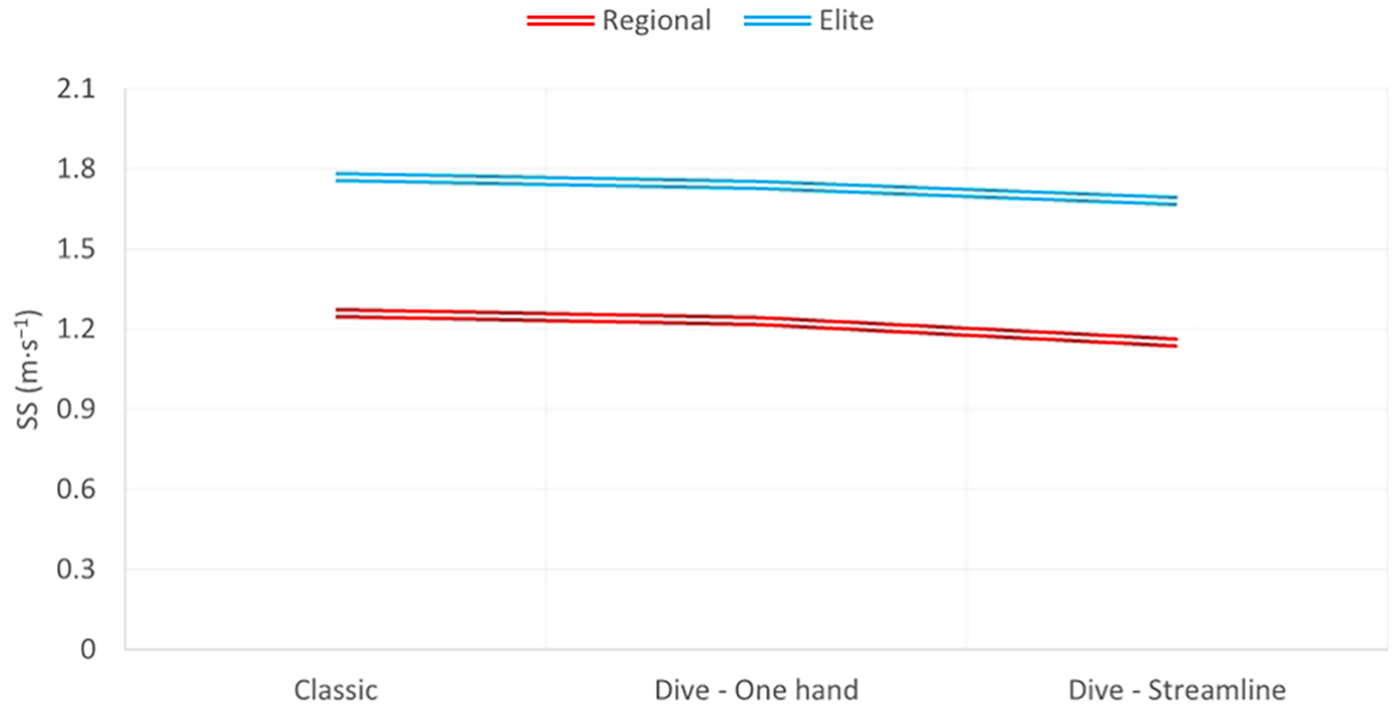
Swimming speed of the last 5 m (finish) between regional and elite-level swimmers in the three types of finish. SS, swimming speed.

Similarly, the SS at the last 5 m was faster on the elite level than the regional level swimmers in all types of finish (*p* = 0.00) (Figure 2). However, there is a downward trend, in the SS, from Classic to Dive–Streamline finish in both elite and regional level swimmers.

**Figure 3:**
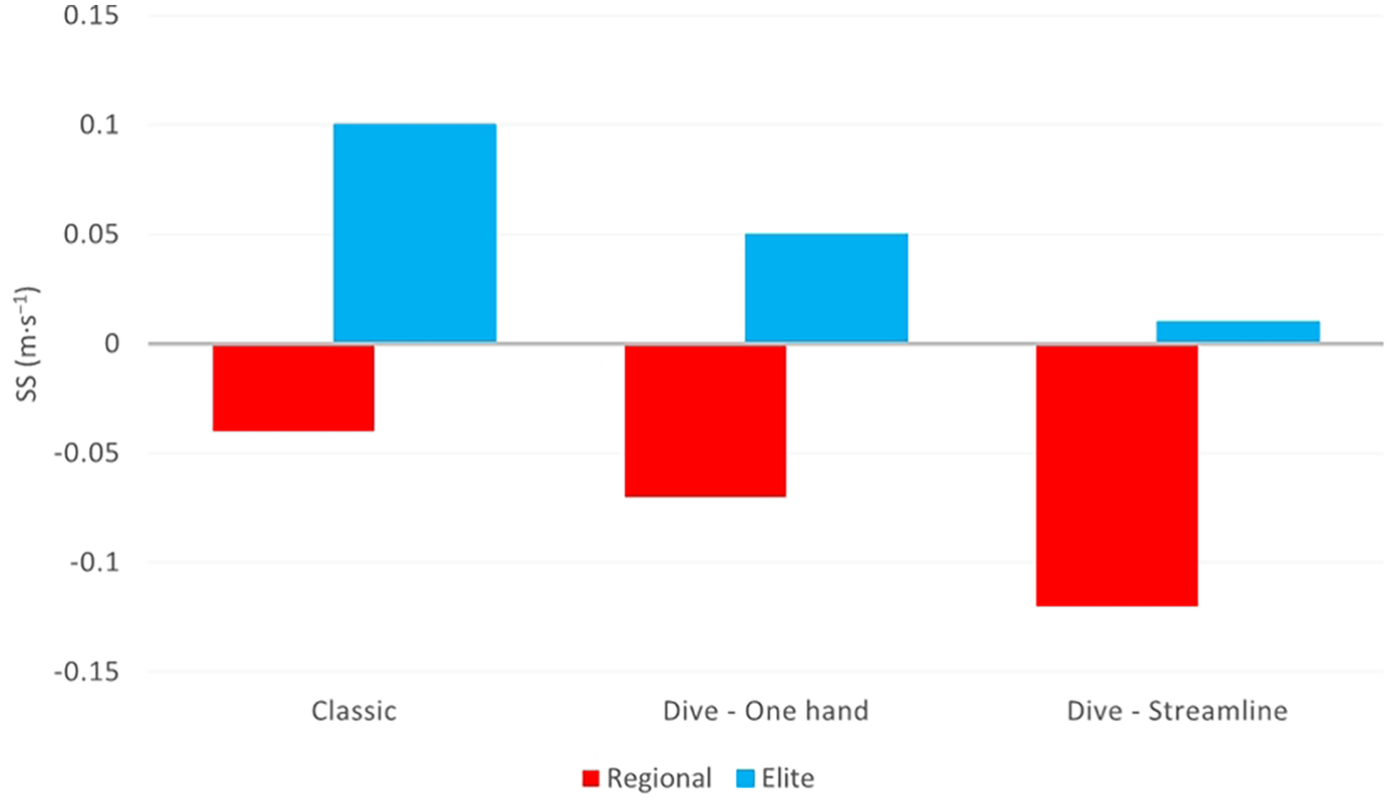
Comparison of swimming speed transition from the intermediate 10 to the last 5 m between regional and elite-level swimmers. SS, swimming speed.

The difference between the SS of the intermediate (10 m) and the last meters (5 m) was positive in the elite swimmer, who increased his SS. On the other hand, the regional swimmers had negative differences showing a deacceleration at the same transition point (Figure 3).

## 4 Discussion

In the present comparative case study, an Olympic-level swimmer implemented the two new backstroke finish approaches (9), as well as regional-level swimmers did in our previous study (8). The measurements indicated that the elite swimmer performed better at the intermediate 10 m compared to the regional-level swimmers, which was in line with the obvious hypothesis based on their difference in World Aquatics (WA) points. However, although the elite swimmer increased his swimming speed (SS) from the intermediate 10 to the last 5 m, compared to the regional-level swimmers, who decelerated during the same transition phase, the significant point is that there was a similar downward tendency between the three backstroke finishes at both levels of swimmers, with the Classic type being the fastest, followed by the Dive–One hand and the Dive–Streamline in the last place.

Generally, dolphin kicks have been shown to enhance swimming performance (18–20). Thus, the implementation of dolphin kicks during the backstroke finish could potentially enhance swimmers' performance. It is well-established that many modifications in the regulations occur for the enhancement of swimmers' performance. A recent example is the downward butterfly kick, which has been implemented during the underwater phase of the breaststroke start and turn (9), providing the opportunity to break the water's surface for the first stroke even further than the first 15 m as in other swimming styles.

In the present study, we attempted to improve our methodology, comparing the deficiencies and limitations of our previous study (8). To do so, we enlisted one of the top national and international-level swimmers (World Aquatics points of 883 ± 64) to participate. This participant's profile was comparable to those in the study conducted by Marinho et al. (20), where elite swimmers competed in the European Championship. Furthermore, we employed a more advanced analytical approach for the last 5 m of the swim. This approach included evaluating factors such as the underwater distance (UD) covered by the swimmer after sinking, the speed from the 5 m line until just before sinking (SBS), the sink-to-finish speed (SFS), which measures the velocity from the head's sink until the hand touches the wall, the head depth (HD), which analyzes the maximum depth of the head during the sink, and the time of head depth (THD), which calculates the time taken for the swimmer's head to reach the maximum depth. As referred, there was a difference in both values of the intermediate 10 m and the last 5 m between the regional and elite-level swimmers (8, 20).

Specifically, the elite swimmer, in Classic, Dive–One hand, and Dive–Streamline finishes, had 0.37–0.40 hundredths of a second faster intermediate SS, while being more efficient (SL, SR, and SI) compared to the regional-level swimmers, which is reasonable considering the differences in the WA points (883 vs. 330) (8), elements of training (volume and intensity), and experience. Moreover, the SS difference, from the intermediate 10 m to the last 5 m (transition phase), was positive for the elite-level swimmer, increasing his velocity from 0.01 to 0.1 m·s^−1^, in contrast to the regional-level swimmers whose SS difference was negative, demonstrating a drop from 0.04 to 0.12 m·s^−1^.

Therefore, the elite swimmer had the experience to manage a faster transition into the last 5 m, indicating the higher level and demands of the races in which he had participated. Both Marinho et al. (20) and Papadimitriou and Papadimitriou (8) regarded the importance of a fast backstroke finish, especially at the elite level. Therefore, the elite swimmer was expected to demonstrate this positive SS transition from the intermediate to the last meters.

Our previous study (8) examined only the SS of the last 5 m, while in the present study, we extended the variables to explore the pros and cons of these two finish variations. Exploring the additional factors, the swimmer in the Dive–One hand and Dive–Streamline finishes needed fewer strokes than in the Classic type (1 vs. 4 strokes), which is reasonable, considering the underwater dolphin kicks that were implemented one stroke after the last 5 m line in the two new variations of the backstroke finish. However, the SBS, HD, and THD seem to be the main contributors to this deceleration in the Dive–One hand and Dive–Streamline.

The excessive drop in SS due to this early sink affects the transition SS possibly because of the instantaneous increase in the body's inclination angle when the swimmer changes from the full stroke to the dolphin kick phase. The result is in line with Papadimitriou and Papadimitriou (8), who explained that the increased frontal area may cause this deceleration, because of the increased body drag, during the HD, which was 24.19 and 20.79 s for the Dive–One hand and Dive–Streamline, respectively. This effect was also depicted through the excessive SBS decrement, which was 0.97 and 0.77 m·s^−1^ for Dive–One hand and Dive–Streamline, respectively.

However, there is the belief that these two backstroke finish variations still have the potential to dominate and be a choice for swimmers. Firstly, in our previous study (8), there was a high correlation between dolphin kick ability and the two new backstroke finishes. In the present study, this hypothesis was confirmed through the SFS factor. More specifically, the faster dolphin kick speed compared to the regional-level swimmers (1.83 vs. 1.06 m·s^−1^, *p* = 0.04) showed an extreme increase in velocity at the SFS, 2.16 and 2.28 m·s^−1^ for Dive–One hand and Dive–Streamline, respectively. However, there are no comparative data from our previous study reflecting this difference.

**Figure 4:**
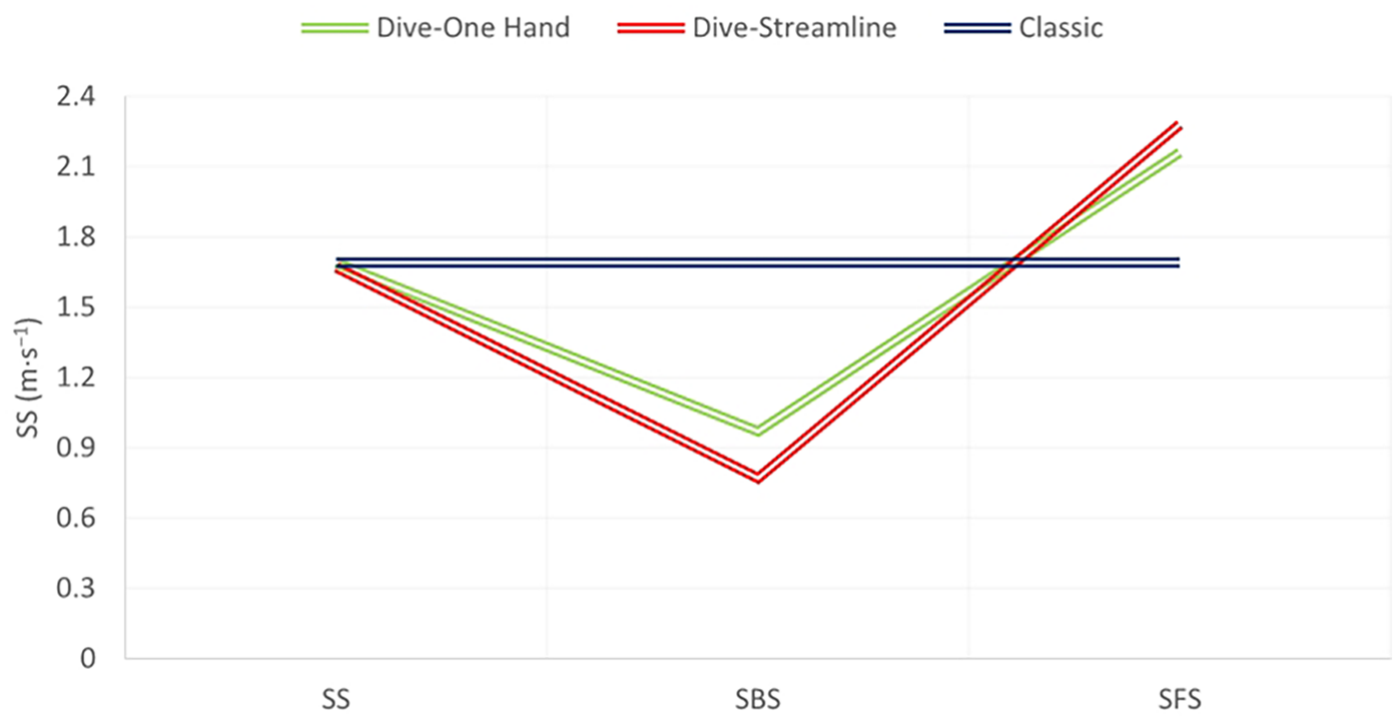
The SS variation between the three measurement points of the backstroke finish. SS: swimming speed in the intermediate 10 m. SBS, speed before sink; SFS, sink to finish speed.

A main contributor to faster dolphin kick ability is kick frequency (8), which depends on the coordination from the torso to the toes (20, 28). The elite swimmer had faster dolphin kick ability than the regional-level swimmers (8, 29). Therefore, swimmers whose average swimming velocity and kick frequency are significantly higher than junior swimmers (18) can benefit from these types of backstroke finishes. Also, by improving the sinking velocity, probably with uninterrupted motion during the transition from swimming to the sink, there is the potential for a faster finish in the Dive–Streamline and Dive–One hand compared to the Classic finish (Figure 4).

Based on the current study's findings, it can be hypothesized that the new variations of backstroke finishes hold the potential for dominance, particularly when swimmers possess a high level of dolphin kick ability. This hypothesis aligns with the findings from the study involving regional-level swimmers. However, due to the important limitation of involving only a single elite swimmer, and consequently, the results cannot be generalized, although we used the Crawford-Howell *t*-test for the comparison between the case and the mean values of a sample. Therefore, further investigations involving more elite swimmers, focusing on differentiating the sinking approach and improving the SBS factor, could potentially provide more insight into the contribution to the performance of these two new backstroke finishes, especially in the 50 and 100 m, where the differences between swimmers are measured in hundredths of a second.

## 5 Conclusions

The elite swimmer can utilize the two variations of backstroke finishes more effectively compared to regional-level swimmers. The swifter dolphin kick ability appears to play a significant role in achieving a successful backstroke finish, while the reduction of SBS seems to be a crucial element for the dominance of these two variations of backstroke finish. Further investigations differentiating the sinking approach, with more elite swimmers' participation, will strengthen the hypothesis that these two new variations of backstroke finish can be effectively utilized by swimmers, particularly those with high dolphin kick speed.

## Data Availability

The raw data supporting the conclusions of this article will be made available by the authors, without undue reservation.
